# Clinical Translation of Three-Dimensional Scar, Diffusion Tensor Imaging, Four-Dimensional Flow, and Quantitative Perfusion in Cardiac MRI: A Comprehensive Review

**DOI:** 10.3389/fcvm.2021.682027

**Published:** 2021-07-07

**Authors:** Sophie Paddock, Vasiliki Tsampasian, Hosamadin Assadi, Bruno Calife Mota, Andrew J. Swift, Amrit Chowdhary, Peter Swoboda, Eylem Levelt, Eva Sammut, Amardeep Dastidar, Jordi Broncano Cabrero, Javier Royuela Del Val, Paul Malcolm, Julia Sun, Alisdair Ryding, Chris Sawh, Richard Greenwood, David Hewson, Vassilios Vassiliou, Pankaj Garg

**Affiliations:** ^1^Department of Cardiovascular and Metabolic Health, Norwich Medical School, University of East Anglia, Norwich, United Kingdom; ^2^Department of Cardiology, Norfolk and Norwich University Hospital, Norwich, United Kingdom; ^3^Department of Infection, Immunity and Cardiovascular Disease, University of Sheffield, Sheffield, United Kingdom; ^4^Multidisciplinary Cardiovascular Research Centre & Division of Biomedical Imaging, Leeds Institute of Cardiovascular and Metabolic Medicine, University of Leeds, Leeds, United Kingdom; ^5^Bristol Heart Institute and Translational Biomedical Research Centre, Faculty of Health Science, University of Bristol, Bristol, United Kingdom; ^6^Cardiothoracic Imaging Unit, Hospital San Juan De Dios, Ressalta, HT Medica, Córdoba, Spain

**Keywords:** cardiovascular magnetic resonance, diffusion tensor imaging, tissue characterisation, myocardial fibrosis, four-dimensional flow imaging

## Abstract

Cardiovascular magnetic resonance (CMR) imaging is a versatile tool that has established itself as the reference method for functional assessment and tissue characterisation. CMR helps to diagnose, monitor disease course and sub-phenotype disease states. Several emerging CMR methods have the potential to offer a personalised medicine approach to treatment. CMR tissue characterisation is used to assess myocardial oedema, inflammation or thrombus in various disease conditions. CMR derived scar maps have the potential to inform ablation therapy—both in atrial and ventricular arrhythmias. Quantitative CMR is pushing boundaries with motion corrections in tissue characterisation and first-pass perfusion. Advanced tissue characterisation by imaging the myocardial fibre orientation using diffusion tensor imaging (DTI), has also demonstrated novel insights in patients with cardiomyopathies. Enhanced flow assessment using four-dimensional flow (4D flow) CMR, where time is the fourth dimension, allows quantification of transvalvular flow to a high degree of accuracy for all four-valves within the same cardiac cycle. This review discusses these emerging methods and others in detail and gives the reader a foresight of how CMR will evolve into a powerful clinical tool in offering a precision medicine approach to treatment, diagnosis, and detection of disease.

## Introduction

Non-invasive imaging plays a fundamental role in the assessment of cardiovascular disease. Cardiovascular magnetic resonance (CMR) is now considered the gold standard imaging technique for the assessment of myocardial anatomy, regional and global function, and viability. More recently, novel methods of CMR are pushing the boundaries of diagnosis and allowing CMR to guide treatment whilst also further sub-phenotyping cardiovascular diseases.

Myocardial tissue characterisation has undoubtedly been proven to be an invaluable tool for clinicians as it often provides the diagnosis, therefore enabling the most appropriate treatment option. Established techniques, including native T1-mapping, extracellular volume (ECV) quantification, and T2-mapping, are used in clinical practise routinely not only to differentiate cardiomyopathies but also to provide information on the extent of myocardial disease as evidenced by oedema and/or fibrosis encountered in several disease processes that affect the myocardium. CMR also allows individualised planning of invasive management strategies against arrhythmias. More specifically, localisation of the scar tissue can provide invaluable information for targeted ablation treatment strategies.

More advanced techniques, such as quantitative perfusion CMR and diffusion tensor imaging (DTI), have shown promising advancements that further support the transition of their use from research to the clinical practise. Moreover, novel methods such as the four-dimensional (4D) flow CMR have revolutionised the field as it allows direct evaluation of the flow in all three directions. The information provided by 4D flow can be vital in the assessment of conditions such as complex valvular lesions or congenital heart diseases, in which the haemodynamic patterns and effects have been difficult to visualise with other imaging modalities.

CMR therefore plays an important role in several aspects of clinical practise having significant impact on the diagnostic, prognostic and treatment pathways in patient care. In this comprehensive review, we first discuss established methods of CMR and then describe important emerging methods which have the potential for clinical impact and are likely to influence the future role of CMR.

## Tissue Characterisation

CMR is now established for myocardial tissue characterisation and allows the assessment of myocardial oedema, microvascular obstruction, thrombus, and scar (1). Clinical use includes—T2-weighted STIR (short tau inversion recovery) imaging for myocardial oedema and intra-myocardial haemorrhage post-acute myocardial infarction (MI), early gadolinium enhancement (EGE) for thrombus and microvascular obstruction and late gadolinium enhancement (LGE) for the myocardial scar. The pattern of the myocardial scar on LGE can sub-phenotype the disease process (2). In addition, a combination of the aforementioned methods can further differentiate acute from chronic MI (3), cause of heart failure (HF) or cardiomyopathy (4, 5). Importantly, visual identification of LGE has been associated with worse prognosis across a wide series of pathologies, including aortic stenosis, cardiomyopathies, and congenital heart disease (6–8). Furthermore, myocardial and liver iron content quantification by T2* magnetic resonance has been well-established in the management of conditions with iron-overload and particularly in the serial follow-up of patients with thalassaemia (9). These standard methods have some limitations—they are predominantly non-quantitative methods, which might limit their standardisation and utilisation as endpoints in clinical trials. Whilst quantification of LGE is being investigated in the research arena, and higher LGE mass has been associated with worse prognosis, this is not being routinely utilised (10, 11). Also, these are two-dimensional acquisitions which limit full left ventricular (LV) coverage for either diagnosis or therapeutic reasons, for example, in ventricular tachycardia (VT) ablation (12). Here we describe emerging CMR tissue characterisation methods that have the potential for improving diagnosis, informing treatment, and outcomes.

## Scar Mapping by LGE Imaging for Arrhythmic Ablation Therapy

**Figure 1:**
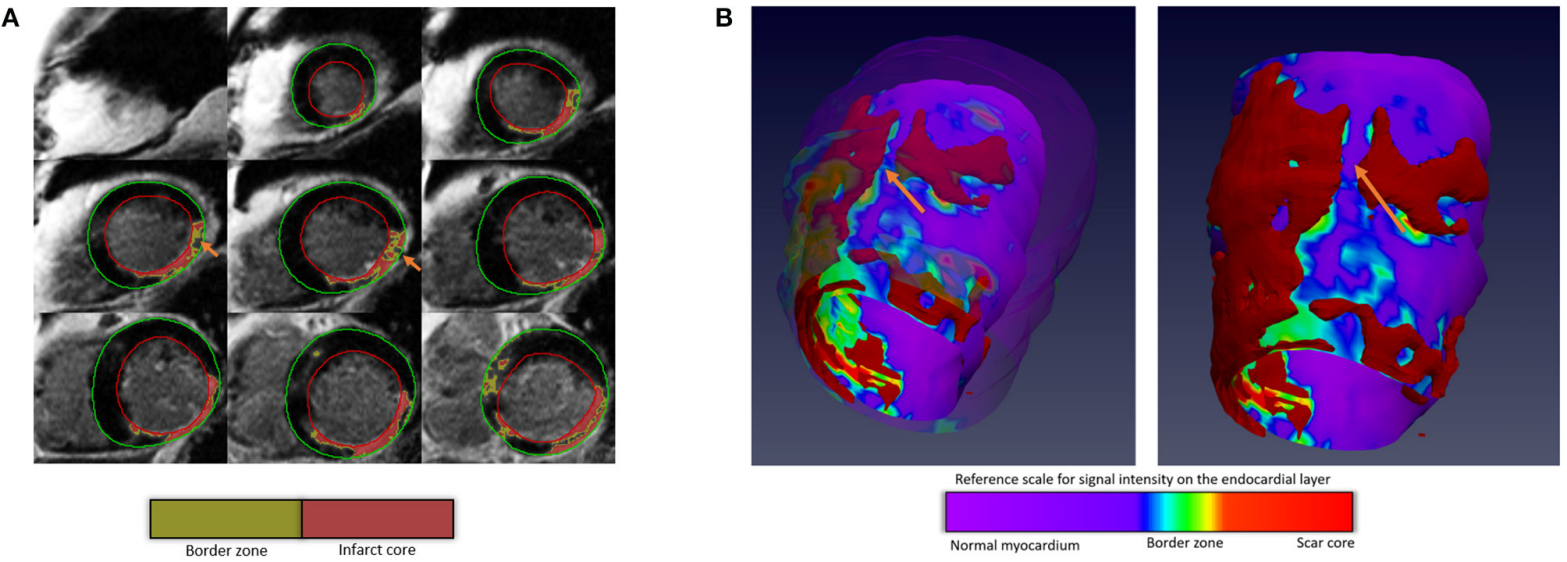
Scar mapping. **(A)** Short-axis, two-dimensional segmentation of scar and border zone. **(B)** These are high-resolution signal intensity maps generated using two 2D LGE acquisitions. Full-width half max threshold of 60% has been applied to identify the infarct core—the red areas. The orange arrow demonstrates the presence of deep channel with late action potential which was ablated for this patient.

The presence of myocardial scar and its characteristics define the arrhythmogenic substrate (13, 14). In particular, the partially infarcted myocardial tissue, named the grey zone, has been shown to be an important predictor of adverse cardiac events, outperforming traditional functional parameters such as left ventricular ejection fraction (15, 16). More recently, the deep channels, islets of alive myocytes sandwiched between two discrete infarcted regions have been identified as areas of late potential, which can be targeted for ablation (Figure 1) (17). Translational studies have already demonstrated that the arrhythmogenic deep channel ablation, identified by using LGE imaging, can reduce arrhythmic burden and recurrence over electrical mapping techniques. In addition, scar localisation (epicardial vs. subendocardial) allows planning for either an endocardial or epicardial approach for ablation. For example, for ablation of interventricular septum ventricular tachycardia, epicardial access is typically not required. This has the huge potential to save time in the catheter laboratories, reduce patient risk and discomfort and overall demonstrate promising outcome results. To map the core scar, grey zone, and the deep channels, a three-dimensional (3D) coverage of the left ventricle or the atrium is necessary. A proposed solution is to acquire short-axis and long-axis stacks of the region of interest and generate a high-resolution 3D stack using this (18). With the emergence of novel acceleration methods, including compressed sensing, it is now possible to do 3D LGE imaging with full coverage of the LV or left atrium (LA) for informing ablation therapy. CMR-aided scar de-channelling results in a lower need for radio-frequency delivery, higher rates of non-inducibility after substrate ablation, and VT-recurrence-free survival (17). However, integrated software solutions are needed to push this emerging method into routine clinical practice.

## Quantitative Tissue Characterisation

**Figure 2:**
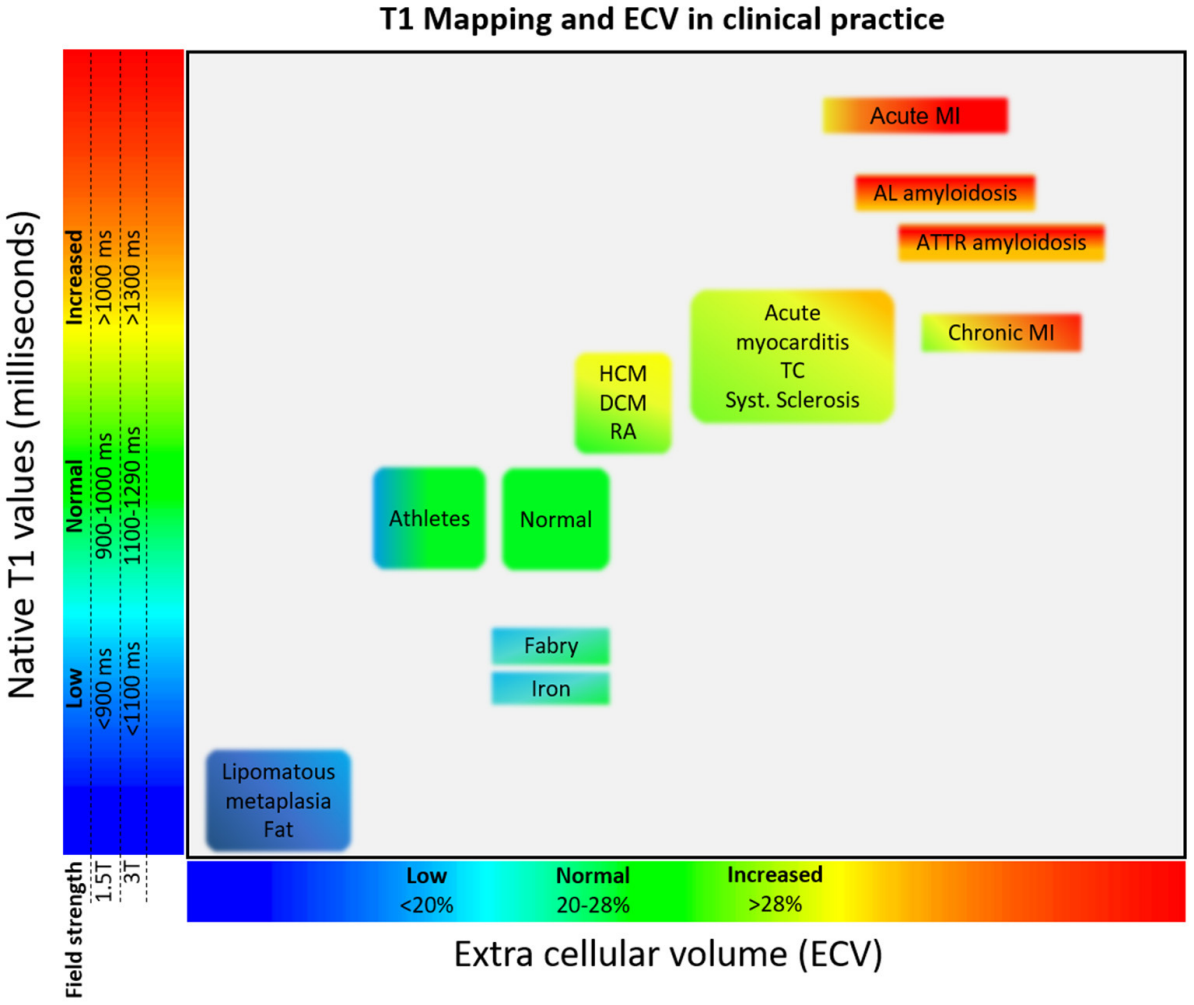
Tissue characterisation using native T1 and ECV. Absolute values for native T1 depend greatly on field strength (1.5 or 3 T), pulse sequence (MOLLI or ShMOLLI), scanner manufacturer and rules of measurements. For the purpose of comparability, only studies using MOLLI sequences were considered in this figure. AL, amyloid light chain; ATTR, amyloid transthyretin; DCM, dilated cardiomyopathy; ECV, extracellular volume; HCM, hypertrophic cardiomyopathy; MI, myocardial infarction; RA, rheumatoid arthritis; TC, takotsubo cardiomyopathy.

**Figure 3:**
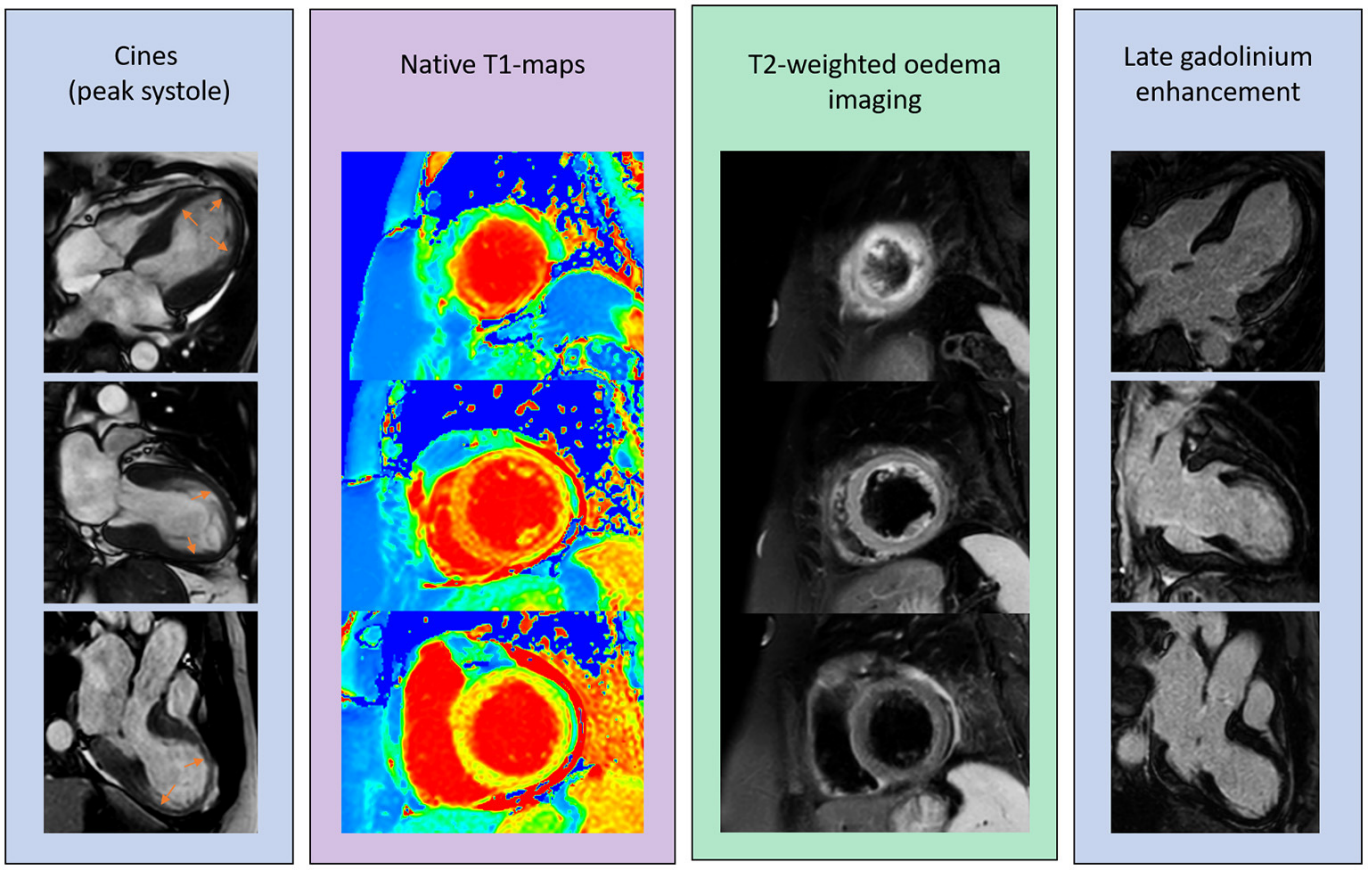
Multi-parametric tissue characterisation of the myocardium to aid diagnosis. This is a case of a 57 year old lady who presents with acute chest pain, ST elevation, and raised troponin biomarkers. Her coronary angiogram was normal. An acute CMR revealed apical ballooning demonstrated in the 4CH view by orange arrows, rise in myocardial oedema on T2-weigthed imaging and confirmed on quantitative native T1-maps (white arrows). There was no evidence of scar on late gadolinium enhancement imaging and overall the extracellular volume was relatively not high as T1-values. This is suggestive of more intra-cellular swelling than extracellular expansion in an acute setting.

**Figure 4:**
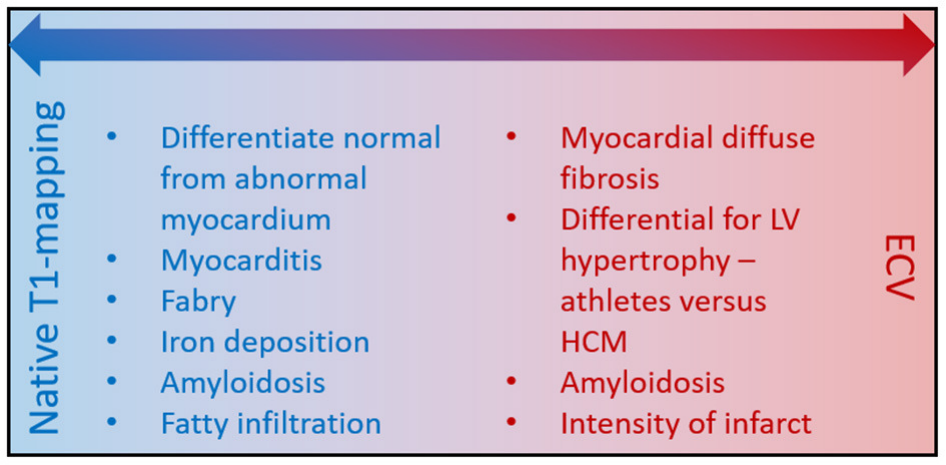
Diagnostic complementary utility of native T1-mapping vs. ECV. The main advantage of ECV is that it takes gadolinium flow kinetics into consideration to improve the precision of diagnosis.

In the last 5-years, several CMR relaxometry-based quantitative techniques have translated into routine clinical practice to inform diagnosis and guide treatment (19, 20). The most common ones are native T1-mapping, extracellular volume (ECV) quantification using native/post-contrast T1-mapping methods, T2-mapping, and T2*-mapping. These techniques are now established to sub-phenotype cardiomyopathies and differentiate the aetiology of myocardial infarction with non-obstructive coronary arteries (MINOCA) in an acute setting (Figure 2) (21). A case example of Takotsubo cardiomyopathy diagnosed acutely on multi-parametric CMR is demonstrated in Figure 3. In addition, native-T1 and ECV mapping allow quantifying infarct size, area at risk, and myocardial salvageable index (MSI) in an acute infarct setting (22, 23). In patients who have contraindications for gadolinium-based contrast agents, native-T1 allows making some degree of infarct assessment; however, as it is less specific, it is not always possible to infer infarct using this method alone. Diagnostic strengths of native T1 vs. ECV are shown in Figure 4.

In recent years, the focus of the assessment and risk stratification of patients with valvular lesions has shifted towards the myocardium. For example, in the case of aortic stenosis, there remains a major uncertainty regarding the timing of intervention in asymptomatic patients. Myocardial fibrosis in aortic stenosis is mainly a result of chronically elevated left ventricular afterload, which is associated with several pathological changes, including cellular hypertrophy, expansion of the extracellular matrix (or volume), and ischaemia due to demand-supply mismatch (24). Several studies have shown that once replacement fibrosis ensues, it progresses rapidly and is unlikely to improve following aortic valve replacement (25, 26), diffuse fibrosis indexed for the left ventricular volume measured by the indexed ECV (iECV) regress after aortic valve intervention (25, 26). Following correction of the afterload issue by valve intervention, the left ventricular mass decreases with regression of both cellular and extracellular mass, but cellular mass regresses more, hence resulting in relative increase in ECV% as the ratio of matrix to total mass is increased. However, the iECV decreases as it represents the extracellular matrix as a total volume, rather than a percentage which is therefore in keeping with the potential for reversal of diffuse fibrosis. Clinical trials, including the EVOLVED trial, are investigating the role of myocardial fibrosis assessment as a trigger for intervention (27). Quantitative tools (T1-mapping and ECV) for mapping diffuse fibrosis are of particular interest in aortic stenosis. There is some evidence that myocardial fibrosis mapped using these tools is associated with histology in aortic stenosis (28, 29); they can detect ventricular decompensation (24), further risk stratification, and predict adverse outcomes offering incremental value to LGE (30, 31).

In the case of the mitral valve, the role of diffuse myocardial fibrosis assessment by mapping techniques is less established. A study by Edward et al. (32) demonstrated the ECV in patients with asymptomatic primary mitral regurgitation to be raised. In addition, ECV was associated with two previously defined important prognosis markers—end-systolic volume and left atrial volume, and with peak VO2 max (*r*-0.51, *P* < 0.05). Furthermore, the impact of diffuse fibrosis on patients symptoms and ventricular response following surgery is being addressed by the FINDER study (33).

## Quantitative Myocardial Perfusion

Vasodilator stress perfusion CMR imaging has evolved into a recognised form of assessment for patients with known or suspected cardiac chest pain for the identification of myocardial ischaemia. Visual assessment involves identification of delayed first-pass wash-in of gadolinium-based contrast agent, from epicardial to endocardial layers in one or more myocardial segments. By comparison with single photon emission computed tomography (SPECT), perfusion CMR allows better assessment of transmural perfusion due to its high spatial resolution and has been shown to be superior in the evaluation of left main stenosis (34) and in assessment of women with suspected ischaemia (35).

First-pass perfusion images are usually acquired by a dynamic T1-weighted sequence; this is meant to generate a contrast between zones of stress-induced hyperaemic myocardium and zones of relatively reduced perfusion, based on the distinct speed of inflow of blood and gadolinium over time. Typically stress images are performed under vasodilator (adenosine, dipyridamole, or regadenoson) stress followed by rest images, which comprise the same sequence without the vasodilator effect, usually as three short axis (basal, mid, and apex) slices. Automated inline quantification enables the user to obtain myocardial blood flow (MBF) at stress and rest. Using the values of stress and rest MBF, a ratio known as the myocardial perfusion can be obtained.

Despite the more widespread availability of CMR in recent years, quantitative perfusion CMR has remained largely a research tool, and perfusion CMR is assessed visually in the clinical setting. Clinical perfusion CMR compares well to invasive angiography (36, 37), fractional flow reserve (FFR) (38–42), and single positron emission computed tomography (SPECT) (43–45), and has demonstrated good prognostic value (46–48).

Over the past three decades, quantitative perfusion CMR has been described (47, 48), refined, and subsequently validated against FFR (40, 49), microspheres (50), and more recently against PET (51, 52). Though PET remains the non-invasive reference method for perfusion quantification, it involves the use of ionising radiation and requires an on-site cyclotron.

The aim of a quantitative approach is to enable user-independent and reproducible measurements of myocardial perfusion. This is especially pertinent in cases where perfusion abnormalities are diffuse as visual assessment can be challenging. In the case of multi-vessel disease, a quantitative approach has been shown to be superior to visual assessment (53). Similarly, in an observational registry, the use of a quantitative approach proved it could provide incremental prognostic benefit over a visual approach (54).

**Figure 5:**
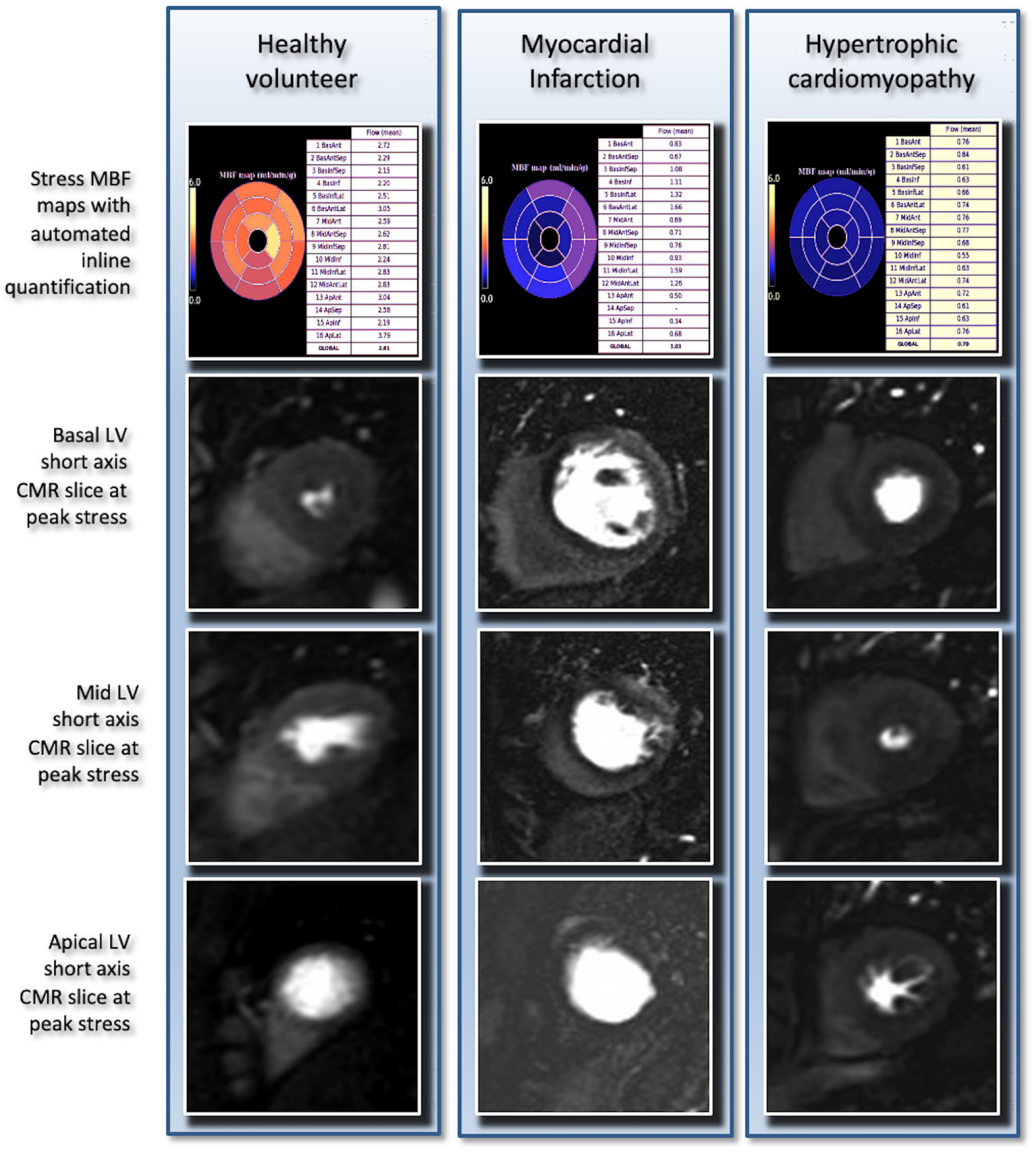
Stress perfusion CMR images obtained by automated in line quantification as well as the basal, mid, and apical slices that were obtained in a healthy volunteer showing no inducible ischaemia, in a patient with a myocardial infarction showing inducible ischaemia in the basal to apical septal and inferior segments and in a patient with hypertrophic cardiomyopathy demonstrating globally reduced myocardial blood flow secondary to severe microvascular disease.

Lack of standardisation appears to be the limiting factor to more widespread implementation of quantitative perfusion CMR particularly in regards to acquisition and dosing protocols, analysis methods, and the availability of software for post-processing across centres. The last few years, however, have seen significant steps towards more user-friendly quantitative approaches to drive towards clinical translation. Using a dual-sequence approach, Kellman et al. have achieved inline myocardial perfusion quantification allowing fast results with minimal user interaction (Figure 5). This approach has been validated in healthy volunteers (55, 56), in patients with known or suspected coronary artery disease (CAD) against angiography (56) and in patients against PET (57). Other recent studies have explored the use of perfusion mapping against angiography (58), deep learning-based processing of perfusion data (59), and most recently, artificial intelligence quantification of perfusion mapping was shown to be a strong, independent predictor of adverse cardiovascular outcome (60).

Perfusion quantification is based on the indicator-dilution principle (61). A recognised amount of an indicator is injected into a fluid flowing at an unknown rate. This is through a system of unknown volume. The fluid is monitored at different points downstream from the plane of introduction and the concentration of indicator, diluted by the parent fluid, is measured as a function of time. In first-pass perfusion, the “indicator” is usually a gadolinium-based contrast agent introduced *via* a peripheral vein. Because the concentration of contrast at the ostium of the coronary arteries is not known, the quantity is estimated by measuring the arterial first-pass time-intensity curve (arterial input function, AIF) of the left ventricle. Finally, myocardial blood flow is observed as a function of the quantified myocardial time-intensity curve (62).

Here lies one of the main hurdles in quantification of perfusion CMR—at lower doses, the dose-signal relationship is linear. However, at higher doses, there is saturation of the gadolinium signal curves, which, if uncorrected, can lead to elevated and non-physiological perfusion estimates. As a result, the bulk of the study on quantitative perfusion research has focused on optimisation of approaches to overcome this challenge by addressing various dosing regimes, acquisition protocols, manipulation of magnetic signal, and complex mathematical processing.

Early studies simply used lower doses of gadolinium to avoid saturation effects in combination with strongly T1 weighted sequences—this overcame saturation at higher doses, but however resulted in low myocardial contrast-to-noise ratio, and poor visualisation of perfusion abnormalities (63–65).

There are currently two main approaches to obtaining perfusion-imaging data that can be both visually assessed and quantified; the dual bolus and dual sequence approaches. The dual-bolus method is a combination of a low-high dosage approach, using two consecutive injections of gadolinium contrast (66–69). In short, this is attained by using a combination of a body weight-adjusted and high-dose main bolus of contrast agent preceded by the injection of a low dose pre-bolus having the same volume of the bolus but only 10% of the concentration of gadolinium. The pre-bolus low dosage injection allows the acquisition of a non-saturated AIF. The main bolus injection produces a desired myocardial signal response for both visual and quantitative assessment. The dual-sequence method is a more recently described alternative to the dual-bolus approach, which has gained momentum in recent years (70–72). In this approach, for each cardiac cycle a low-resolution image, with a short saturation- recovery time after the R-wave is acquired to measure the AIF. This avoids any AIF saturation. Low-resolution acquisition is followed by high-resolution acquisition with longer saturation-recovery time to measure the myocardial signal with higher contrast. There are pros and cons associated with both methods; the dual-bolus protocols are feasible in the majority of centres and do not require new software or hardware, but the preparation of the scan is more complex. Conversely, the dual-sequence protocols are more straightforward to implement but widespread use is limited by availability across different scanner vendors.

Following the acquisition of suitable data by either dual bolus or dual-sequence methods, the data must then be analysed to provide stress and rest perfusion estimates and then in turn myocardial perfusion reserve. Myocardial perfusion reserve is arguably more robust as inherent errors within the mathematical modelling are cancelled out by using a ratio between stress and rest. The mathematical approaches proposed are beyond the scope of this review but all rely upon model-dependent or independent deconvolution processing of the AIF and myocardial signals to determine myocardial perfusion (63, 73–84).

Data can be analysed on both a segmental or voxel-wise basis. When segmental quantitative analysis is performed from high-resolution images, the spatial resolution of the images is only partially used, as the signal intensity is sampled transmurally. Hence, time-intensity curves from subendocardial ischaemic areas are averaged. This results in partial-volume effects and possibly reduced sensitivity. High-resolution, voxel-wise myocardial perfusion assessment offers additional information on the heterogeneity of myocardial perfusion (50, 80). This combination of absolute quantification and voxel-level resolution has the potential for calculation of the true ischaemic burden. Hence voxel perfusion maps limit partial volume effects and the influence of other factors such as the LV wall thickness, LV remodeling, and presence of previous infarction.

High-resolution, voxel-wise perfusion is therefore likely to be the more robust method for assessment of ischaemic burden, especially when coupled with full LV coverage enabled by newer 3D perfusion sequences. Though not widely employed, some have demonstrated that 3D quantitative perfusion analysis is feasible with good diagnostic accuracy for CAD (85–88).

Hautvast et al. (89) proposed an alternative approach to exploit and quantify high-resolution perfusion CMR data for the assessment of transmural gradients. This is based on the quantification of perfusion gradients between endocardial and epicardial myocardial layers. The transmural perfusion gradient (TPG) is defined as the percentage of transmural redistribution of myocardial blood flow between layers. The optimal diagnostic threshold for TPG is 20%, which identifies the presence of haemodynamically significant ischaemia in comparison with FFR (90) with high sensitivity, specificity, and diagnostic accuracy. There are several advantages to transmural perfusion gradient analysis compared to other methods of quantitative analysis. Firstly, administration of a diluted pre-bolus is not required nor is the acquisition of rest perfusion images. The method is also more robust to image homogeneity due to variations in the B1 field, different coil configurations, different schemes of contrast agent administration, field strength, and the acquisition pulse sequence (89, 90).

More recently, another approach, the perfusion dyssynchrony analysis, has been proposed (91). This examines both the spatial and temporal dispersion of myocardial signals. The concept rests on the knowledge that in normal myocardium with preserved vasodilatory reserve, the myocardium is perfused uniformly across all segments. The presence of one or more coronary stenoses influences not only the peak but also the time to peak myocardial signal. Specifically, in cases of multi-vessel CAD, the temporal dispersion has been shown to increase in relation to the number of diseased vessels, using FFR as a correlate for haemodynamic significance.

In summary, robust quantification of perfusion has been a goal of many investigators for over two decades. Significant advances in image acquisition, dosing regimens, and analysis methods, particularly in recent years, along with robust validation against a number of techniques, have significantly advanced the field towards clinical translation.

## Diffusion Tensor Imaging

DTI, also known as diffusion tractography, is an advanced CMR technique which allows the study of 3-dimensional whole-organ tissue microstructure, it was initially developed for static organs such as neurological tracts in the brain (92, 93). In more recent years cardiac DTI has become a reality (94). The myocardium has a complex microarchitecture. This consists of a three-dimensional functional syncytium of branching and inter-connecting myocytes. These myocytes are embedded in a collagen matrix. Myocytes are grouped in layers of 5–10, separated by collagen sheetlets which interconnect with neighbouring layers (95, 96). In the left ventricle, the overall direction of myocyte aggregates follows a helical arrangement running in opposite directions at subendocardial and subepicardial levels; the helix is right-handed (positive angulation) at the subendocardium and transitions to being left-handed (negative angulation) at the subepicardum with myofibre aggregates in the mid LV layer being orientated transversely (97). The structure and interplay of the myocytes are intrinsically linked to cardiac contractility and efficiency and therefore the opportunity to perform cardiac DTI offers the potential for deeper understanding of cardiac mechanics in health and disease.

The base principle of DTI involves the imaging of the diffusion of water. In brief, when water is not bound to tissue, it will diffuse at the same rate in all directions—this can be visualised as a sphere. Whereas in a tissue, water will diffuse predominantly in one direction more than in others—this is known as the phenomenon of anisotropy. This results in a different diffusion characteristic that can be seen to progress in different directions and this diffusion property of water can be used to create a map of diffusion. This can be applied to a region of interest—the displacement probability of diffusing water molecules within the myocardium can be measured and the arrangement of the mean direction of water diffusion can be reconstructed. In cardiac imaging, DTI is acquired as a series of two-dimensional short axis ventricular slices which are then reconstructed in 3D form. The use of diffusion-sensitising sequences allows calculation of a set of three eigenvectors with water diffusing more readily along the myofiber, here the average local myocyte orientation within the voxel is represented by the first, largest eigenvector (E1). The second largest eigenvector (E2) corresponds to the average local sheetlet direction. The mean helix angle is represented by E1A. E1A is the angle relative to the local wall tangent plane. E2A represents the mean intra-voxel sheetlet angle. The “third” eigenvector is the sheet-normal that is perpendicular to the helix and sheet plane (also described as the transverse angle). Scalar metrics such as mean diffusivity (MD) and fractional anisotropy (FA) allow phenotyping the structural integrity by registering the degree of free diffusion in a tissue as a general measure. The “diffusivity” is described as the average of the sum of eigenvalues whereas “fractional anisotropy” is described as an index which reflects the degree of anisotropy within a voxel (the variation by which the tissue diffusion contrasts to an idealised sphere).

Early *ex-vivo* cardiac DTI work was demonstrated that cardiac DTI was feasible without destruction of the myocardial architecture and also confirmed that the first and second eigenvectors correlated with histological orientation of the myofibres and sheetlet direction, respectively (98–100). Early pilot studies also explored changes in fibre architecture in pathologies such as myocardial infarction (101, 102). There are several limitations associated with *ex-vivo* cardiac DTI regarding the method/ duration of fixation of the tissue, the cardiac phase in which the heart was fixed (i.e., in a systolic or diastolic state), and the absence of normal loading conditions. *Ex-vivo* work suggested a dynamic rearrangement of the myocytes during contraction, but in recent years, the development of *in-vivo* dual-phase cardiac DTI has built on this. Recent work has shown that macroscopic LV hypertrophy is associated with sheetlet angle during contraction (103, 104).

The application of DTI to a moving tissue, in particular the cardiovascular system, is more challenging than in a static organ. The relaxation time of the myocardium is shorter than in the cerebral tissue, which imposes significant limitations on the echo time that can be used. Moreover, inhomogeneity of the B0-field is increased in the thoracic cavity, particularly at higher field strengths in comparison with the brain. This results in a higher incidence of artefacts, which can affect the diffusion-weighted images. Furthermore, DTI in a beating heart with respiratory motion and displacement of the myocardium during contraction is challenging in that the diffusion of water is several orders of magnitude smaller than bulk motion. The sequences that are used for *ex-vivo* imaging are highly motion-sensitive and therefore are not necessarily suitable for *in-vivo* imaging. A number of other novel DTI sequences have been proposed. The most broadly used technique is the dual-gated stimulated echo acquisition mode (STEAM) sequence. In brief, these involve three excitation pulses applied over two successive heartbeats. This STEAM sequence is relatively insensitive to cardiac motion however it is not robust in arrhythmia. Cardiac DTI is also affected by cardiac strain. The strain produced by myocardial deformation has an impact on diffusion measurements. Proposed approaches to circumvent this issue include imaging at the so-called “sweet-spot” which is a time point, individual for a subject, where the temporal mean of strain approaches zero, thereby eliminating the effect of strain, typically at the mid-systolic or mid-diastolic phase of the cardiac cycle (105, 106). Post-processing methods also allow to correct for the effects of strain (107).

*In vivo* cardiac DTI has reported reproducibility and normal ranges of helix and sheetlet angles in healthy hearts (94, 108–111), and has since been used to explore a variety of pathologies including myocardial infarction, cardiac amyloidosis, and both dilated and hypertrophic cardiomyopathy (112–117). These more recent studies have underlined the potential for DTI to investigate architecture-related changes in disease in relation to biomechanics and over time. Data from *in-vivo* dual-phase cardiac DTI in patients with DCM combined with tagging data and biomechanical modelling explored the relationship between geometry and helix and sheetlet angle, concluding that helix and sheetlet angle changes are likely to be maladaptive rather than compensatory (117). A fascinating and elaborate study by Nielles-Vallespin et al. (113) shed more detailed light on changes in architecture in DCM and HCM; the authors were able to conclude that the myocardium in hypertrophic cardiomyopathy is permanently in a more systolic, contracted state whereas in dilated cardiomyopathy the myocardium is in a more diastolic state. This may potentially account for the predominant diastolic impairment seen in hypertrophic cardiomyopathy compared with the more prevalent systolic impairment seen in dilated cardiomyopathy. Recent studies have also investigated the changes in infarcted tissue comparing DTI with late gadolinium enhancement imaging demonstrating changes in infarcted tissue, suggesting there may be an opportunity to use DTI to evaluate myocardial scar in place of using a gadolinium-based contrast agent (118), and suggested that cardiac DTI may even provide complementary information on remodelling post-myocardial infarction (119). Technical developments in cardiac DTI are ongoing with recent studies exploring the use of cardiac DTI at 7-Tesla (120).

There is no doubt that there is a crucial interplay between the cardiac microstructure, and the biomechanical and electrical functions of cardiac function and that cardiac DTI holds promise in developing a deeper understanding of this relationship. Recent years have seen significant developments in the cardiac DTI, allowing a greater understanding of cardiac structure and function in both health and disease. Though cardiac DTI remains a research tool with ongoing work in optimisation of the technique, in due course this advanced technology may play a part in identifying novel biomarkers or response to new therapies.

## Four-Dimensional Flow

**Figure 6:**
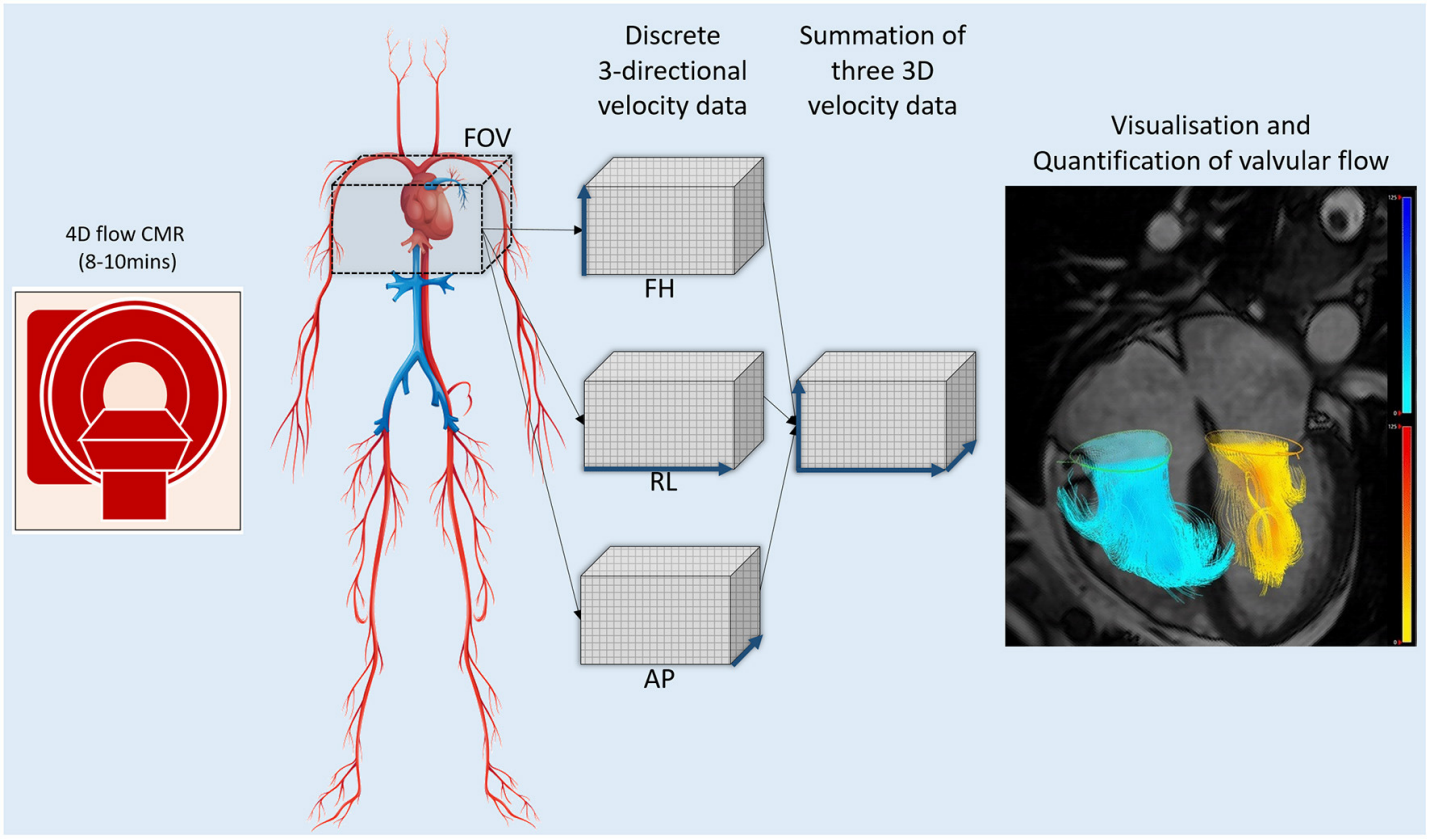
Four-dimensional flow CMR acquisition.

Intracardiac blood flow and its velocities have played a very important role in cardiovascular assessment. However, novel imaging methods like four-dimensional flow CMR (4D flow CMR) imaging are resulting in a paradigm shift as they inform us about flow in all three-directions (Figure 6) (121). We already know that the blood flow is complex, dynamic, and has a diverse 3D profile (122). The fourth dimension in 4D flow CMR imaging is time. More recently, 4D flow CMR has seen significant development allowing for whole-heart cross-sectional flow imaging in under 10 min (123–125).

### Visualisation

**Figure 7:**
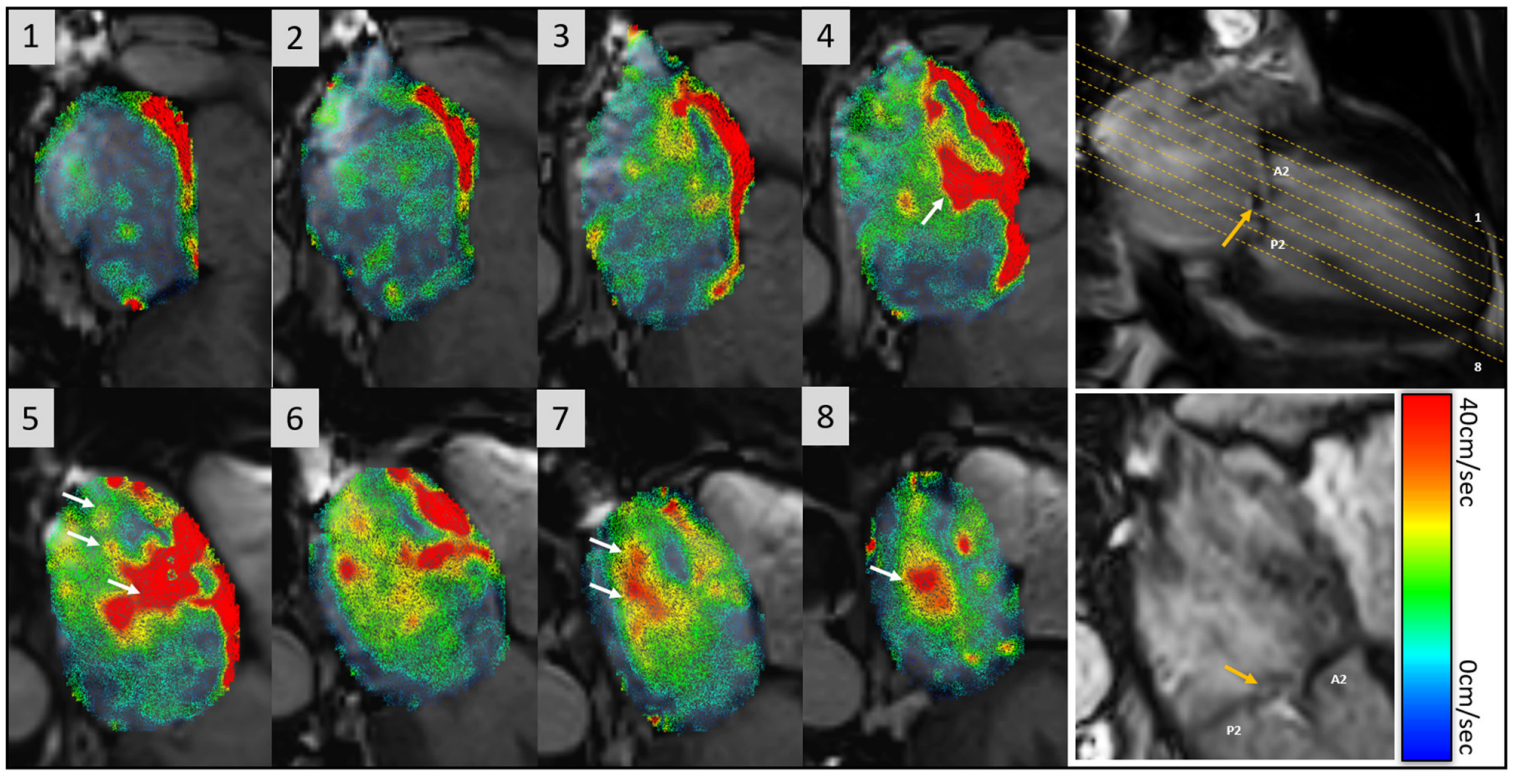
Overview of flow visualisation in the left atrium in a patient with mitral regurgitation secondary to P2 prolapse (orange arrow). 4D flow data is co-registered with cines to demonstrate 2-directional vectors, which have speed encoded colour coding (0–40 cm/s). There is eccentric mitral regurgitation that is directed towards the intra-atrial septum and swirls back into the left atrium (white arrows in panels 4–8). The mitral regurgitation jet almost fills the entire left atrium.

**Figure 8:**
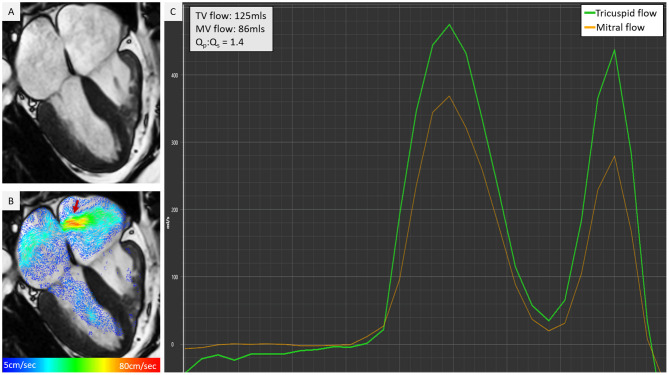
Cine co-registered 4D flow visualisation can offer instant understanding of intra-cardiac shunts and other similar cardiac pathologies. In this figure, there is atrial septal defect that was not clearly recognised on cine **(A)**, however, with 2-directional vector visualisation in **(B)**, there was an obvious left to right shunt. This was quantified by retrospective valve tracking method and significant Qp, Qs was noted **(C)**.

One method to visualise 4D flow is to co-register the velocity coded 2-dimensional flow over the cine planes. This provides an instant and quick assessment of flow vectors depending on the cine visualised (Figure 7). For example, a four-chamber or two-chamber cine can then be used to visualise mitral regurgitation jets using 4D flow CMR. Furthermore, 4D flow CMR enables the visualisation of flow accelerations through stenotic valvular or vascular lesions and can provide additional information in challenging cases such as congenital heart disease where haemodynamic effects of anatomical abnormalities are not always clear. 4D flow CMR visualisation can be integrated into routine CMR reporting and can add diagnostic advantage. Valvular stenotic flow accelerations and pathological eccentricity of the flow can be determined by visualisation of 2-directional velocity vectors. This becomes clinically relevant, for example, an eccentric aortic ejection flow direction would raise the suspicion of bicuspid aortic valve or aortic stenosis (126, 127). Routine visualisation of 2-directional vectors on four-chamber cine can enhance detection of septal defects, flow acceleration in outflow tracts, and valvular regurgitation (Figure 8).

### Vortex Imaging

We are now developing a better understanding of blood flow behaviour inside the ventricles using 4D flow CMR. Vortices are formed in almost all chambers of the heart and major vessels. Vortices can be due to optimum physiological flow (128), pathological flow due to dilatation (129), or raised pressures (130). Vortical flow in the main pulmonary artery is an example of pathological flow and is associated with elevated main pulmonary arterial pressure (131). Vortical blood flow in the main pulmonary artery tends to be pathological and a threshold >14.3% of the cardiac interval is associated with pulmonary hypertension (sensitivity: 97%; specificity: 96%). Similarly, in aortic root dilation, more helical flows with vortices have been demonstrated as pathological (132). Another study also showed that flow abnormalities may be a major contributor to aortic dilation in patients with bicuspid aortic valve disease (133).

### Quantification of 4D Flow

4D flow CMR not only helps to visualise but also allows to quantify vascular, valvular, and intra-ventricular flow. These include transvalvular flow, intra-cavity velocity or kinetic energy (KE) assessment, vortex quantification, blood flow component analysis, and haemodynamic forces quantification (134). From the above-mentioned methods, valvular flow quantification using retrospective valve tracking is ready for clinical adoption as it has been validated and has demonstrated superior accuracy to standard 2D phase-contrast methods (134–136). The other method which shows promising intra-/inter-observer reliability is intra-cavity blood flow KE mapping (137). Most of the other techniques remain research tools, which have offered mechanistic insight into several disease processes and are currently under investigation of direct clinical applications.

### Quantification of Transvalvular Flow

**Figure 9:**
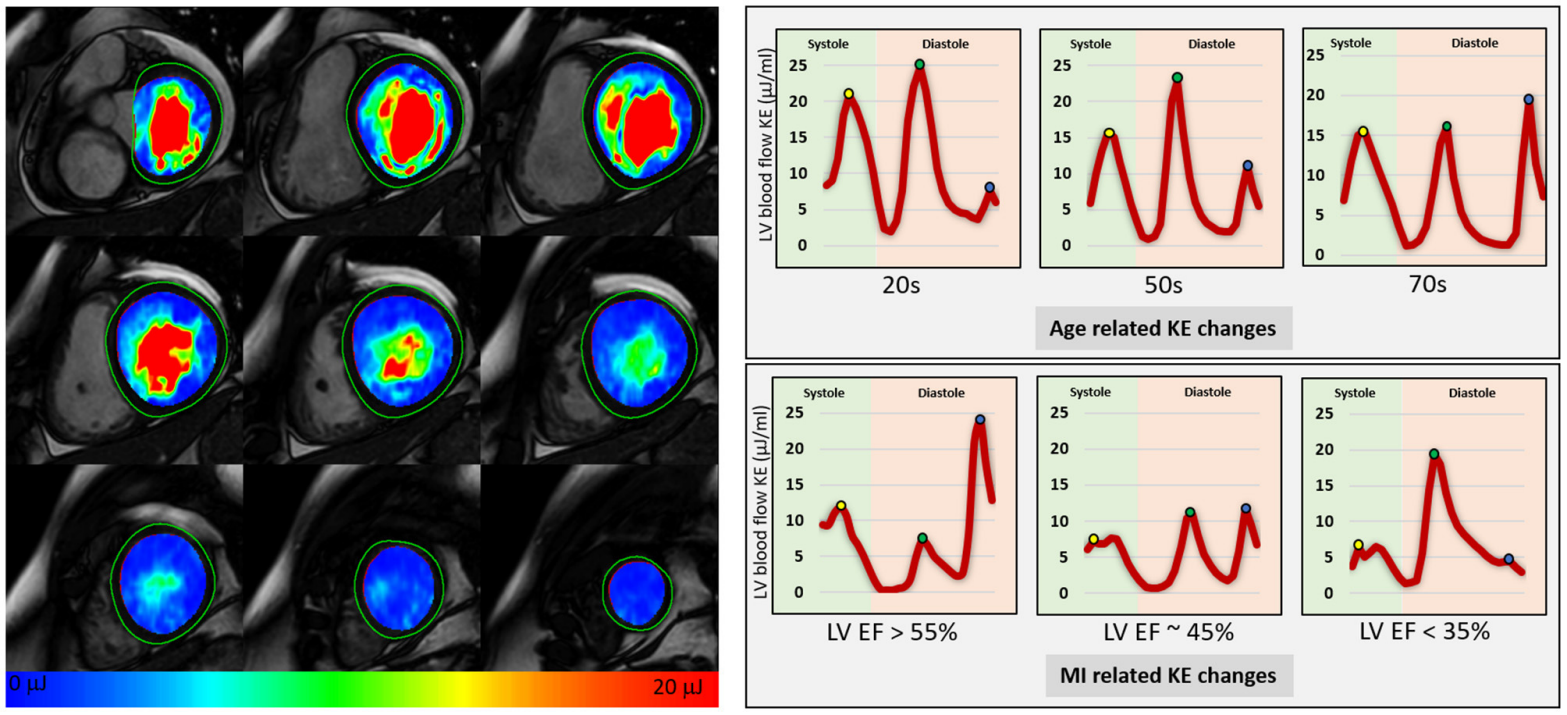
Left ventricular blood flow kinetic energy mapping in healthy, ageing, and in patients with myocardial infarction.

4D flow CMR in the context of retrospective valve tracking allows quantification of flow through all four heart valves for the same averaged cardiac cycle, whilst factoring in valve motion by tracking the valve (138) (Figure 9). This valve tracking method overcomes the limitations of conventional 2D phase-contrast velocity encoded imaging or Doppler ultrasound-based through-plane motion. Therefore, 4D flow CMR flow quantification methods can be considered to be the non-invasive “gold-standard” for intracardiac flow quantification (139–141). In patients with limited apical ultrasound views, 4D flow CMR may provide an alternative to assess peak E and A velocities for LV diastolic function assessment. Moreover, 4D flow CMR can be used to directly quantify regurgitant volumes by generating a reformatted plane in the 4D volume data set that is exactly perpendicular to the regurgitant jet (142). Software solutions with automated valve tracking procedures reduce post-processing time significantly and are becoming more readily available (143).

### Quantification of Blood Flow Kinetic Energy

4D flow CMR also allows us to quantify the KE of blood flow through a chamber of the heart for the complete cardiac cycle. The KE of a moving blood flow with mass (m) and velocity (v) is KE = ½m × v^2^. In this equation mass is (blood viscosity) × (voxel size). Using the KE formula, the whole left ventricular blood flow KE can be quantified by summing the KE of each individual voxel within the ventricular cavity. This is achieved by superimposing the time-resolved endocardial contours defined from cine images onto the 4D flow data to extract velocity information. The analysis of KE is predominantly automated. As this technique uses endocardial contours which have established high reproducibility, a key strength of KE quantification is its excellent intra-/inter-observer agreement and almost instant haemodynamic assessment without needing any additional post-processing (137). As LV blood flow KE takes into consideration complete intra-cavity flow, it is a better marker of diastolic function when compared to standard diastolic parameters (Figure 9) (137). Furthermore, studies have shown how it is an independent predictor of adverse remodelling post-myocardial infarction (144) and even LV thrombus formation (145). This emergent tool holds great potential for phenotyping cardiac haemodynamics, informing diagnosis, and potentially predicting disease course. Future trials are needed to evaluate its value in informing treatment outcomes in cardiovascular diseases.

## Future Perspectives

The role of CMR and its inclusion in multiple guidelines across the breadth of cardiology has been expanding steadily in recent years. Whilst conventional techniques provide without doubt the gold standard assessment for anatomy, function, and tissue characterisation in a non-invasive radiation-free method, the emergence of novel methods of CMR will allow incremental value not only in the diagnosis, but also in the management of more patients with cardiac pathology. It is only a matter of time before the described techniques become more broadly available to the cardiovascular imaging community and further prognostic studies validate the utility of these techniques for clinical translation.

## Conclusion

CMR has a role of paramount importance in the assessment and diagnosis of cardiovascular disease. We reviewed the conventional CMR methods currently used in clinical practice but also described novel methods likely to enter the clinical arena in the future. With these novel methods, CMR can successfully guide clinical management further by revealing clinically important clues related to cardiac anatomy and function. Technical developments, advanced methods of image acquisition, and a better understanding of cardiac haemodynamics provide the foundation for further research and validation of the newly emerging tools and techniques that are being proved to be of extreme clinical importance.

## Author Contributions

SP, VT, HA, and BM: original draft preparation, writing - review and editing, reference management, and quality checks. PG, AC, PS, AS, and EL: conceptualisation, data curation, and project administration. PG, AC, ES, AD, JC, and JR: figures, illustrations, and content. JS, AR, CS, RG, PM, DH, VV, and PG: critical appraisal, editing, and draft final version. All authors contributed to the article and approved the submitted version.

## Conflict of Interest

PG is an advisor for Pie Medical Imaging and Medis Medical Imaging. The remaining authors declare that the research was conducted in the absence of any commercial or financial relationships that could be construed as a potential conflict of interest.
